# Brain activity measured by functional brain imaging predicts breathlessness improvement during pulmonary rehabilitation

**DOI:** 10.1136/thorax-2022-218754

**Published:** 2022-12-26

**Authors:** Sarah L Finnegan, Michael Browning, Eugene Duff, Catherine J Harmer, Andrea Reinecke, Najib M Rahman, Kyle T S Pattinson

**Affiliations:** 1 Nuffield Department of Clinical Neurosciences, University of Oxford, Oxford, UK; 2 Oxford Health NHS Foundation Trust, Warneford Hospital, NHS, Oxford, UK; 3 Department of Psychiatry, University of Oxford, Oxford, UK; 4 Department of Paediatrics, University of Oxford, Oxford, UK; 5 Oxford Respiratory Trials Unit, University of Oxford, Oxford, UK; 6 Oxford Chinese Academy of Medicine Institute, University of Oxford, Oxford, UK; 7 Oxford NIHR Biomedical Research Centre, University of Oxford, Oxford, UK; 8 Nuffield Division of Anaesthetics, Oxford University Hospitals NHS Foundation Trust, Oxford, UK

**Keywords:** Pulmonary Rehabilitation, COPD epidemiology, Perception of Asthma/Breathlessness, Imaging/CT MRI etc

## Abstract

**Background:**

Chronic breathlessness in chronic obstructive pulmonary disease (COPD) is effectively treated with pulmonary rehabilitation. However, baseline patient characteristics predicting improvements in breathlessness are unknown. This knowledge may provide better understanding of the mechanisms engaged in treating breathlessness and help to individualise therapy. Increasing evidence supports the role of expectation (ie, placebo and nocebo effects) in breathlessness perception. In this study, we tested functional brain imaging markers of breathlessness expectation as predictors of therapeutic response to pulmonary rehabilitation, and asked whether D-cycloserine, a brain-active drug known to influence expectation mechanisms, modulated any predictive model.

**Methods:**

Data from 71 participants with mild-to-moderate COPD recruited to a randomised double-blind controlled experimental medicine study of D-cycloserine given during pulmonary rehabilitation were analysed (ID: NCT01985750). Baseline variables, including brain-activity, self-report questionnaires responses, clinical measures of respiratory function and drug allocation were used to train machine-learning models to predict the outcome, a minimally clinically relevant change in the Dyspnoea-12 score.

**Results:**

Only models that included brain imaging markers of breathlessness-expectation successfully predicted improvements in Dyspnoea-12 score (sensitivity 0.88, specificity 0.77). D-cycloserine was independently associated with breathlessness improvement. Models that included only questionnaires and clinical measures did not predict outcome (sensitivity 0.68, specificity 0.2).

**Conclusions:**

Brain activity to breathlessness related cues is a strong predictor of clinical improvement in breathlessness over pulmonary rehabilitation. This implies that expectation is key in breathlessness perception. Manipulation of the brain’s expectation pathways (either pharmacological or non-pharmacological) therefore merits further testing in the treatment of chronic breathlessness.

**WHAT IS ALREADY KNOWN ON THIS TOPIC**

- Pulmonary rehabilitation is an effective treatment for many, but not all people with chronic obstructive pulmonary disease (COPD who suffer from chronic breathlessness in COPD. Baseline patient characteristics predicting improvements in breathlessness are unknown.

**WHAT THIS STUDY ADDS**

- This is the first study to identify a model capable of predicting changes in breathlessness over pulmonary rehabilitation at the individual patient level. The study shows that prerehabilitation breathlessness expectation related brain activity is a strong predictor of clinical improvement in breathlessness over pulmonary rehabilitation.

**HOW THIS STUDY MIGHT AFFECT RESEARCH, PRACTICE OR POLICY**

- Manipulation of the brain’s expectation pathways (either pharmacological or non-pharmacological) merits further testing in the treatment of chronic breathlessness.

## Introduction

Chronic breathlessness is a key feature of chronic obstructive pulmonary disease (COPD) with symptoms often persisting despite maximal medical therapy. Pulmonary rehabilitation is the best treatment for chronic breathlessness in COPD^1^ but the response is variable. Thirty percent of people who complete pulmonary rehabilitation derive no clinical benefit.^2^ Despite considerable research, we still do not know which patient characteristics predict beneficial response to pulmonary rehabilitation.^2–5^ The ability to predict outcome has a number of potential benefits. These include improving our understanding of underlying mechanisms, identifying targets for personalised medicine which may allow more accurate allocation of scarce healthcare resources.

Breathlessness severity is often poorly explained by objective clinical measures.^6^ This has prompted research into identifying brain perceptual mechanisms that may explain this discordance. A body of work has recently identified that brain processes relating to expectation (akin to placebo and nocebo effects) have an important role in contributing to breathlessness severity. Whether brain-derived metrics may help predict outcome from pulmonary rehabilitation is unknown, and prediction models until now have not included measures of expectation.

Between-subject variability in therapeutic response is increasingly recognised as a confounder in clinical trials. A personalised medicine approach aims to identify subgroups of patients that respond to a specific therapy. In psychiatry, brain-derived metrics using functional neuroimaging have taken similar approaches to identifying subtypes of depression that may respond to bespoke therapies.^7^ Such techniques rely on biomarkers, which may consist of predictive combinations of biochemical, genetic, demographic, physiological or cognitive measures. In the context of treating breathlessness, predictive biomarkers could pave the way for novel pharmacological and non-pharmacological treatments. These may either work as stand-alone therapies, or by enhancing other therapies, such as pulmonary rehabilitation.

In this study, we aimed to predict improvements in breathlessness during pulmonary rehabilitation by analysing baseline data from a longitudinal experimental medicine study of D-cycloserine on breathlessness during pulmonary rehabilitation. We selected D-cycloserine, which is a partial agonist at the NMDA receptor in the brain, for its action on neural plasticity and influence on brain expectation mechanisms associated with cognitive behavioural therapies.^8–10^ Brain-based pharmacological adjuncts may be one opportunity to boost the effects of pulmonary rehabilitation. We hypothesised baseline brain activity in response to breathlessness-related expectation would predict improvement in breathlessness over pulmonary rehabilitation, and that if D-cycloserine indeed had an effect on expectation then it would emerge as a significant factor in the prediction model. Given that moderators of treatment success of pharmacological agents such as D-cycloserine remain unclear, this information will help build a better picture of the brain-behaviour changes that may underly response to pulmonary rehabilitation and therefore clarify its potential value as a therapeutic target.

## Methods and materials

A brief overview of materials and methods is presented here with full details included within online supplemental materials. The study and statistical analysis plan were preregistered on bioXIV (https://osf.io/bfqds/). This was an analysis of data from a longitudinal experimental medicine study of patients with COPD over a course of pulmonary rehabilitation. Parts of the study were first published in a characterisation of baseline patient clusters^11^ and subsequently in the investigation of the effect of D-cycloserine on brain activity (preprint https://doi.org/10.1101/2021.06.24.21259306). The analysis conducted for this study is novel, not previously reported and is the first use of predictive analysis using this dataset.

10.1136/thorax-2022-218754.supp1 Supplementary data

### Participants

Seventy-one participants (18 female, median age 71 years (46–85 years)) (online supplemental table 1) were recruited immediately prior to enrolment in a National Health Service-prescribed course of pulmonary rehabilitation. Full demographic details are included within online supplemental materials and are published separately (preprint is available at https://doi.org/10.1101/2021.06.24.21259306).

### Study protocol

Data for this analysis were acquired at baseline assessment held at the start of a pulmonary rehabilitation course, and following completion of the pulmonary rehabilitation at 6–8 weeks. At each study visit, identical measures were collected. Following the first visit, participants were randomised in a double-blind procedure to receive either 250 mg oral D-cycloserine or a matched placebo. Participants received a single dose on four occasions 30 min prior to the onset of the first four pulmonary rehabilitation sessions.

#### Self-report questionnaires

All questionnaires (table 1) were scored according to respective manuals: Dyspnoea-12 (D12) Questionnaire,^12^ Centre for Epidemiologic Studies Depression Scale,^13^ Trait Anxiety Inventory,^14^ Fatigue Severity Scale,^15^ St George’s Respiratory Questionnaire,^16^ Medical Research Council (MRC) breathlessness scale,^17^ Breathlessness catastrophising scale, adapted from the Catastrophic Thinking Scale in Asthma,^18^ Breathlessness vigilance, adapted from the Pain Awareness and Vigilance Scale Breathlessness Awareness and Vigilance Scale.^19^

**Table 1:** List of measures included within each of the three models (indicated by ‘X’)

Included data	Brain only model	Brain and non-imaging measures model	Non-imaging measure model
Drug ID	x	x	x
Responder or non-responder label	x	x	x
Brain activity			
Amygdala	x	x	
Hippocampus	x	x	
Anterior insula	x	x	
Anterior cingulate	x	x	
Posterior insula	x	x	
Putamen	x	x	
Superior marginal gyrus	x	x	
Superior frontal gyrus	x	x	
Precuneus	x	x	
Medial prefrontal cortex	x	x	
Caudate	x	x	
Posterior cingulate	x	x	
Angular gyrus	x	x	
Precentral gyrus	x	x	
Middle frontal gyrus	x	x	
Questionnaires			
D12		x	x
CES-D		x	x
TRAIT		x	x
FSS		x	x
SGRQ		x	x
MRC		x	x
BCS		x	x
Vigilance		x	x
Physiology			
FEV1/FVC		x	x
MSWT—HR change		x	x
MSWT—SPO2 change		x	x
MSWT—distance		x	x
MSWT—BORG change		x	x
BMI		x	x
Pack-years		x	x
Age		x	x
Sex		x	x

Drug ID labels corresponded to whether the participant received D-cycloserine or placebo.

BCS, breathlessness catastrophising scale; BMI, body mass index; BORG, rating of perceived exertion; CES-D, Centre for Epidemiologic Studies Depression Scale; D12, Dyspnoea-12; FEV1, forced expiratory volume; FSS, Fatigue Severity Scale; FVC, forced vital capacity; HR, heart rate; MRC, Medical Research Council; MSWT, Modified Shuttle Walk Test; SGRQ, St George’s Respiratory Questionnaire; SPO2, oxygen saturation; TRAIT, Trait Anxiety Inventory.

#### Physiological measures

Spirometry and two Modified Shuttle Walk Tests (MSWT) were collected using standard protocols.^20 21^ Participant height and weight were recorded at each visit. Arterial oxygen saturations were collected at rest and following the MSWT.

### MRI measures

#### Image acquisition

MRI of the brain was carried out using a Siemens 3T MAGNETOM Trio. A T1-weighted (MPRAGE) structural scan (voxel size: 1×1 × 1 mm) was collected and used for registration purposes. A T2*-weighted, gradient echo planar image (EPI) scan sequence (TR, 3000 ms; TE 30 ms; voxel size: 3×3×3 mm) was used to collect functional imaging data during the word cue task.

#### Word cue task

Given sufficient fearful breathlessness exposures, the suggestion alone of the situation can be sufficient to drive a top-down neural cascade and produce breathlessness in the absence of afferent inputs. We drew on this link to probe the neural responses of breathlessness-related expectation by examining the activity of brain regions responding to breathlessness-related word cues.^22 23^ Brain activity was correlated with corresponding visual analogue ratings of breathlessness and breathlessness-anxiety collected during scanning. During the fMRI scanning, participants were presented with a word cue, for example, ‘climbing stairs’ in white text on a black background for 7 s. Participants were then asked, ‘how breathless would this make you feel’ (wB) and ‘how anxious would this make you feel’ (wA). To each question participants responded within a 7 s window using a button box and Visual Analogue Scale. The response marker always initially appeared at the centre of the scale, with the anchors ‘not at all’ and ‘very much’ at either end. Scan duration was 7 min and 33 s.

## Analysis

### Regions of interest

**Figure 1:**
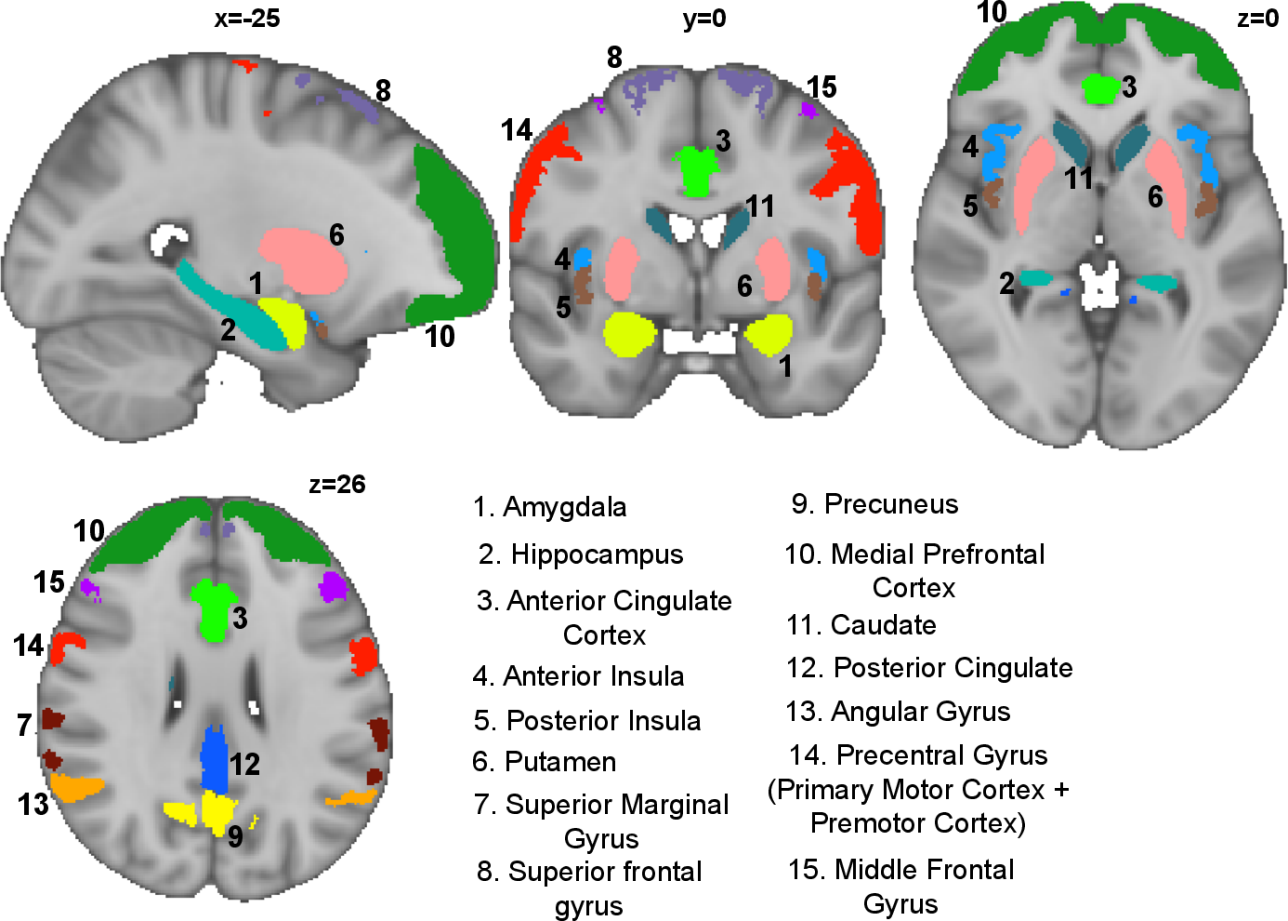
Region of interest map showing 15 brain areas.

Fifteen regions of interest were selected a priori (figure 1), encompassing regions associated with sensory and affective processing of breathlessness as well as body, symptom perception and emotional salience.^22 24 25^ Regions were defined by standard anatomical atlas maps (Harvard-Oxford Atlas and Destrieux’ cortical atlas), thresholded at 40% probability and binarised.

### Brain imaging analysis

Image processing was carried out using the Oxford Centre for Functional MRI of Brain Software Library (FMRIB, Oxford, UK; FSL V.5.0.8; https://ww.fmrib.ox.ac.uk/fsl/), MATLAB R2018b (Mathworks, Natick, Massachusetts, USA) and associated custom scripts. Functional MRI processing was performed using FEAT (FMRI Expert Analysis Tool, within the FSL package).

### Preprocessing and single subject models

Data were preprocessed according to standard protocols which included motion correction and physiological noise removal, before being entered into single subject general linear models. These models captured brain activity during the periods in which the breathlessness-related word cues were presented allowing us to examine expectation-related processes (online supplemental figure 3). Further details regarding preprocessing and model specifics can be found within online supplemental materials. For each participant, the mean signal in response to the breathlessness-related word cues was extracted for each brain region (figure 1). This gave each participant 15 brain-derived scores to enter into the predictive models.

### Definition of response to pulmonary rehabilitation

Responsiveness to pulmonary rehabilitation was defined as a change in D12 score, a well-validated clinical measure of breathlessness, of three or more points, consistent with the minimal clinically important difference.^12^ To examine whether baseline D12 score differed significantly between responders and non-responders to pulmonary rehabilitation we conducted a comparison of average baseline D12 score. In addition, a single logistic regression model (table 2) was applied to the data using MATLAB’s mnrfit function to examine whether baseline D12 explained pulmonary rehabilitation outcome over and above the best model prediction. Significance was set as false discovery rate corrected p<0.05.

**Table 2:** Logistic regression coefficients for predictive power of the computationally derived brain-behaviour model (model prediction labels), and baseline D12 on pulmonary rehabilitation outcome, measured as a change in D12 score above the minimal clinical important difference

	Coefficient	P value
Intercept	3.571	0.007
Model prediction labels	−2.809	<0.001
Baseline D12	0.071	0.191

Significance is expressed as false discovery rate (FDR)—corrected p values.

D12, Dyspnoea-12.

### Predictive models

#### Physiological measures

Spirometry and two MSWT were collected using standard protocols.^20 21^ Participant height and weight were recorded at each visit. Sex was self-reported. Arterial oxygen saturations were collected at rest and following the MSWT.

**Figure 2:**
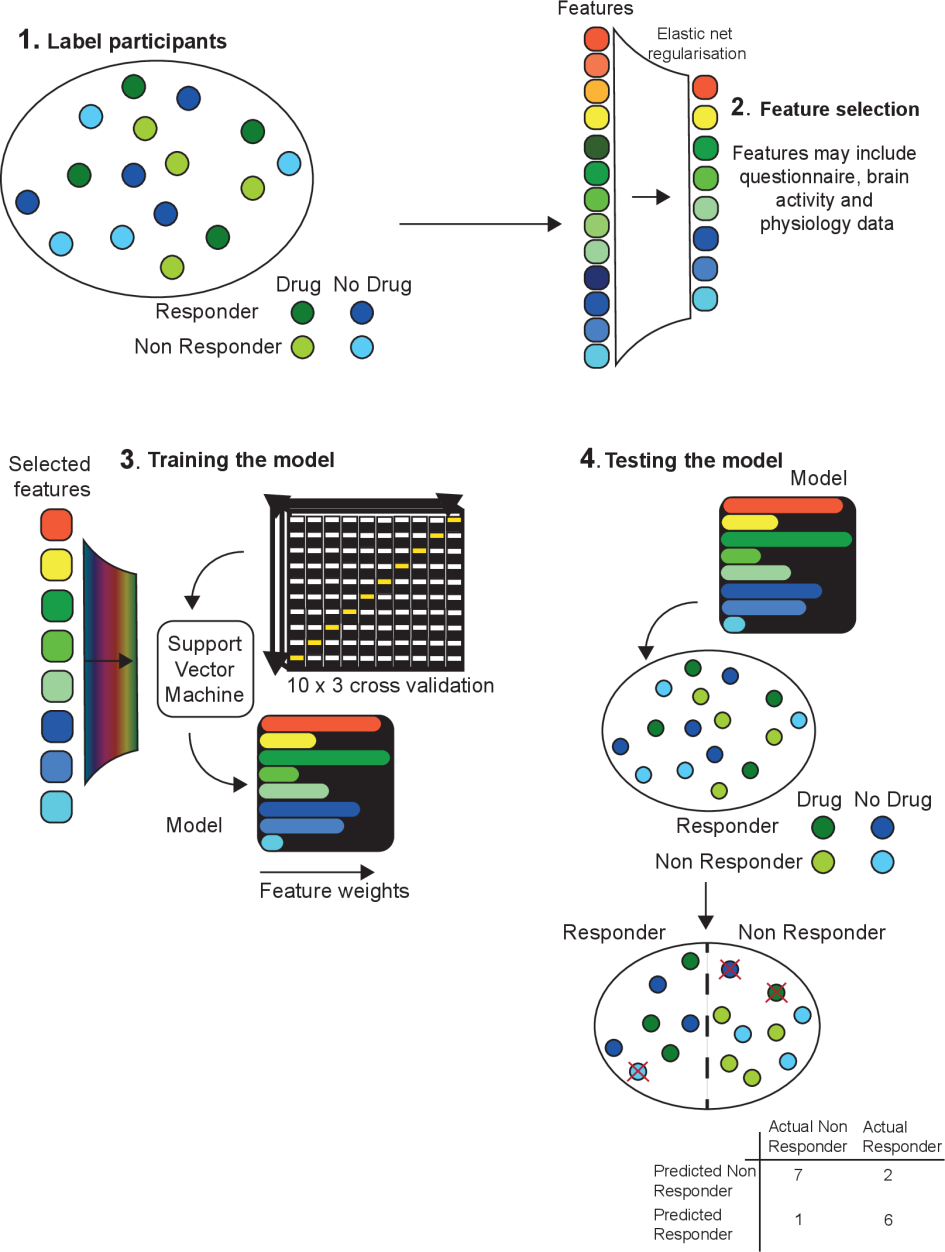
A schematic of the modelling procedure adapted from an illustration by Chekroud and colleagues.^37^ (1) Participants received two labels, the first corresponding to drug or placebo and the second ‘responder’ or ‘non-responder’ to treatment. (2) An elastic net procedure was used to rank and select the top features. (3) Selected features were used to develop model training parameters in a repeated-cross validation procedure in which the algorithm never has access to the entire dataset. (4) The Trained SVM was then provided with the entire dataset to classify. In addition to model statistics, full confusion matrices were output to assess sensitivity and specificity.

Models were programmed using R V.3.6.1 (2019-07-05). Modelling procedure remained the same for each of the three models (figure 2).

1. All measures were centred and scaled. Checks were performed to determine whether any measures were highly correlated (R>0.8) or linear combinations of each other.
2. To correct for imbalance in the number of responders/non-responder a resampling procedure. Imbalanced classes can affect classifier performance. Random OverSample Examples (ROSE) was carried out. ROSE, an R package, creates an artificially balanced sample using a smoothed bootstrap approach.^26^
3. An elastic net procedure was used to identify the number most relevant features for inclusion into the model. Elastic net procedure was selected for its ability to regularise, improve data sparsity via feature selection and cluster correlated measures together (for more details see online supplemental materials). Features were selected based on ranked coefficients.
4. Model training parameters—C, kappa and sigma were selected based on an internal repeated cross-validation procedure (10-fold cross validation repeated 3 times). In all instances automated tuning parameter selection for the values, with a tune length of 5, was used within R’s caret package. Train test data were kept separate across folds, with the algorithm never having access to the entire dataset. The best tuning parameters were selected automatically by R’s caret package from across cross validation folds.
5. These parameters were used to train a Support Vector Classifier with radial kernel to predict outcomes in the entire dataset.
6. Model performance was assessed internally using accuracy, sensitivity, specificity and area under the curve. Full confusion matrices are presented along with calibration curves. Model significance was assessed with a one-tailed binomial test of model accuracy compared with the null information rate.

## Results

### Participants

At baseline the median MRC breathlessness score of the 71 participants was 3 (IQR 1), median FEV1/FVC (forced expiratory volume/ forced vital capacity) was 0.55 (IQR 0.15), median FEV1% predicted was 58 (IQR 21).

### Responders and non-responders

A total of 41/71 participants in the primary dataset met the criteria of a change in D12 score of three or more points to be considered a responder^12^ (24 D-cycloserine, 17 placebo), and 30 did not (13 D-cycloserine, 17 placebo). No significant interaction between responders and non-responders and drug was identified using χ^2^ analysis ((1, N=71) = 1.6, p=0.21). Group changes to D12, St Georges and MSWT are presented in table 3 and online supplemental table 3.

**Table 3:** Average scores on questionnaire and behavioural measures before and after pulmonary rehabilitation

	Comparison of scores before and after pulmonary rehabilitation			
	Before pulmonary rehabilitation	After pulmonary rehabilitation	Uncorrected p values	Corrected p values
D12	10.8 (10.5)	6.5 (7.2)	<0.001	<0.001
MSWT distance (m)	341 (260)	379 (300)	<0.001	0.001
St George—Active	62.5 (27.6)	57.0 (30.9)	<0.001	0.001
St George—Impact	31.1 (20.2)	25.7 (24.4)	<0.001	<0.001
St George—Symptom	61.4 (25.0)	54.9 (28.3)	<0.001*	<0.001*

Variance is expressed as IQR.

Significance is reported as exploratory uncorrected p values and as family wise error (*p<0.05) corrected values.

D12, Dyspnoea-12; MSWT, Modified Shuttle Walk Test.

### Feature selection: brain imaging only model

The elastic net procedure identified 13 of 15 brain-derived metrics and drug as relevant for model inclusion (table 1). These features were: expectation-related brain activity within amygdala, caudate, prefrontal cortex, hippocampus, superior frontal gyrus, anterior insula, drug, posterior cingulate cortex, putamen, posterior insula, middle frontal gyrus, precuneus, precentral gyrus and angular gyrus.

### Feature selection: brain and non-imaging measure model

The elastic net procedure identified 12 of 15 brain-derived metrics, 13 of 20 non-imaging measures including drug as relevant for model inclusion (table 1). These features were brain activity within: superior frontal gyrus, hippocampus, angular gyrus, superior marginal gyrus, amygdala, prefrontal cortex, precuneus, anterior cingulate cortex, anterior insula, middle frontal gyrus, posterior insula and putamen. Behavioural features identified as relevant for model inclusion were D12, anxiety, depression, MRC, the three St George’s domains (active, impact, symptoms), MWST BORG, heart rate and SpO_2_ change, fatigue, age and body mass index.

### Feature selection: non-imaging measures model

Of the 20 questionnaire and physiological features available, only D12 survived the feature selection process (table 1).

### Model results: internal validation

Three models with variables selected by the elastic net procedure were assessed for their ability to discriminate responders from non-responders (table 4).

**Table 4:** Model statistics for brain imaging only, brain and non-imaging measure models and non-imaging measures only model

	Brain only full model	Brain and non-imaging measures full model	Non-imaging measures full model
Accuracy	0.70	0.83	0.66
95% CI	58 to 81	0.72 to 0.90	0.54 to 0.77
Sensitivity	0.93	0.88	0.68
Specificity	0.40	0.77	0.20
AUC	0.79	0.87	0.70
P value	0.02*	<0.001**	0.09

Confusion matrices		Reference		Reference		Reference	
		Yes	No	Yes	No	Yes	No
Prediction	Yes	38	18	36	7	28	11
	No	3	12	5	23	13	19

All models contained drug ID as an additional term.

P value is expressed as the result of a one-tailed binomial test of model accuracy compared with the null information rate.

*p<0.05

AUC, area under the curve.

**Figure 3:**
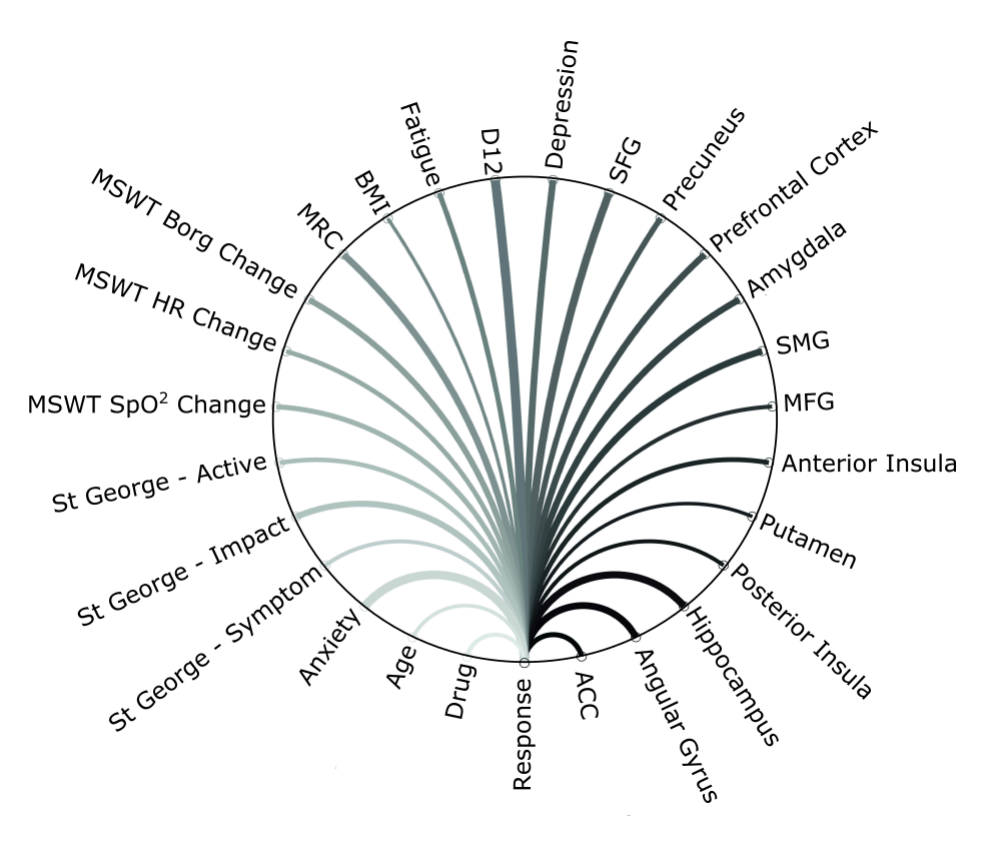
Schematic representation of the best predictive model. Predictive brain imaging and non-imaging measures are shown linked to treatment response by weighted lines, indicating variable importance. ACC, anterior cingulate cortex; BMI, body mass index; D12, Dyspnoea-12; HR, heart rate; MRC, Medical Research Council; MFG, middle frontal gyrus; MSWT, Modified Shuttle Walk Test; SMG, supramarginal gyrus; SpO2, oxygen saturation.

The combination of brain and behaviour metrics produced the best classification performance (accuracy—0.83 (95% CI 0.75 to 0.90); sensitivity—0.88; specificity—0.77; p<0.001) and was well calibrated (table 4, (online supplemental figure 6). Weighted variable importance was found to be similar across features, as demonstrated by the thickness of the lines in figure 3. The brain only model was able to correctly categorise participants with statistically significant likelihood (accuracy 0.70 (95% CI 0.58 to 0.81) but demonstrated poor goodness of fit (online supplemental figure 6).

## Discussion

### Key findings

Using supervised machine learning, this study successfully identified markers that predict clinically relevant improvements in breathlessness over a course of pulmonary rehabilitation. The best model combined brain-imaging markers of breathlessness-expectation, self-report questionnaires and physiology measures, and demonstrated high sensitivity and specificity. Whether or not a participant received D-cycloserine was a significant feature in this model. Our findings demonstrate the first predictive model of change in breathlessness across pulmonary rehabilitation and, for the first time, the clinical relevance of expectation-related brain activity as a therapeutic target in the treatment of breathlessness.

To date, no study has produced a model capable of predicting an individual’s change in breathlessness over pulmonary rehabilitation from baseline traits.^2–4^ Although previous studies have shown correlations between baseline variables and outcomes,^5^ none have attempted to predict outcomes at an individual level. This study is therefore the first to directly predict an individual’s change in breathlessness over pulmonary rehabilitation. This was achieved using sensitive brain imaging techniques in order to capture personalised responses to breathlessness expectation which has, until recently remained relatively unexplored.

Expectation has been linked with symptom severity across conditions including breathlessness and pain,^27 28^ and is well recognised to underly the placebo and nocebo effects. An example of the nocebo effect in breathlessness is provided by a study of healthy volunteers in which, using a conditioning paradigm, a harmless odour was initially paired with induced breathlessness. Subsequently, the odour alone was shown to drive brain activity in the periaqueductal grey and anterior cingulate cortex leading to breathlessness despite the absence of afferent respiratory input.^28^ In Abdallah *et al*,^29^ expectation-related brain activity was associated with poorer responses to opioids in breathlessness, potentially explaining why clinical trials of opioids in the management of breathlessness have been unsuccessful.^30 31^

Fear and anxiety are key components of expectation, which recent research suggests may play a key role in the mechanisms and maintenance of breathlessness.^22 32 33^ Despite this, expectation-related effects have not previously been considered in prediction studies of pulmonary rehabilitation outcome. Our previous work showed a clear correlation between expectation-related brain activity in areas that include the anterior insula, anterior cingulate cortex and prefrontal cortex, and improvements in breathlessness over pulmonary rehabilitation.^22^ However, while these studies suggest baseline cognitive state may be a therapeutically relevant target, importantly, the methods employed so far did not attempt to predict the response of an individual. Taken together, converging lines of evidence point towards expectation-related processes as a clear potential therapeutic target.

In this study, we focused on brain activity changes within a set of pre-selected regions of interest associated with breathlessness-expectation and body and symptom perception.^22 24 25^ In the original trial, we hypothesised that D-cycloserine would augment the therapeutic effects of pulmonary rehabilitation across this network, via its effects on neural plasticity and promotion of expectation-related learning.^10 34^

Using data-driven techniques, 13 of the 15 brain-derived metrics (and drug) were identified as relevant for model inclusion. Selected brain areas spanned the components of relevant body and symptom perception and emotional salience networks. The resulting brain-only model, while statistically significant (p=0.02) and possessing good sensitivity (0.93), did not distinguish responders from non-responders with sufficient specificity (0.40).

By, enriching the brain-only models with questionnaire and physiology measures improved performance considerably. In this enriched model, 12 brain-derived metrics and 13 non-imaging-derived metrics, which included self-report questionnaire measures, physiology and drug, were identified as relevant for model inclusion. Measures of accuracy (0.83), sensitivity (0.88) and specificity (0.77) all suggest this model was able to significantly (p<0.001) predict pulmonary rehabilitation outcome.

Within the non-imaging measure only model, D12 alone was selected by the elastic net and was not found to be significantly (p=0.09, sensitivity=0.68, specificity=0.20) predictive of pulmonary rehabilitation outcome. No other of the 13 non-imaging-derived metrics available was found to contribute to the model. That only D12 was selected suggests that the remaining measures, which were important predictors of rehabilitation outcome in the enriched model, interact strongly with brain activity. These results highlight the value of approaching breathlessness from a multimodal data perspective.

The retained brain activity features implicate a range of brain networks encompassing functions of cognitive control, symptom perception and sensory integration. Activity within these regions has been shown to predict outcome to cognitive behavioural therapy in social anxiety disorder^35^ and obsessive–compulsive disorder.^36^ In our paradigm, in which patients were shown breathlessness related word-cues, triggering expectation related processes, activity within cognitive control network may indicate the allocation of attentional resources. The retained questionnaires within the brain and non-imaging measure model together highlight another important domain of breathlessness: symptom perception. We suggest that baseline symptom perception may act to directly influence the interpretation of the new experiences of pulmonary rehabilitation as positive or negative. At scale, functional neuroimaging is not practicable as a prerehabilitation screening tool, both in terms of cost and access. We do not therefore propose that patients undergo functional neuroimaging as a diagnostic test. However, the use of functional neuroimaging in the research setting can enhance our understanding of how breathlessness may be targeted. For example, the retained brain activity features implicate the allocation of attentional resources, symptom perception and sensory integration. Thus, functional neuroimaging is able to provide insights into the relationship between brain activity and behaviour that is not possible with other techniques. Building on this information, new behavioural therapies may seek to specifically target these domains in parallel or prior to pulmonary rehabilitation.

D-cycloserine has been shown to augment changes to expectation, boosting the therapeutic effects in trials examining anxiety, post-traumatic stress disorder and other mental health conditions,^8–10^ where acute dosages administered prior to exposure-based therapies appear to variously and significantly reduce self-report symptoms of acrophobia, improve clinical symptoms of panic disorder, reduce threat response to fearful faces and associated decision-making reaction times. As a drug which acts on expectation-related brain activity pathways it is therefore not surprising that whether a participant received D-cycloserine or placebo was a retained as a feature in both the brain only model, and to a lesser extent the brain and non-imaging model.

### Limitations and future work

The major limitation of this study is the lack validation of the model in an external dataset. While some studies hold out a proportion of the original data to create an external validation dataset, this technique was not possible here due to restrictions of sample size. To address these limitations, we used a cross validation approach to provide an indication of out of sample transferability in which the support vector machine was exposed to multiple iterations of the sub-sampled dataset during model training, and therefore never ‘saw’ the entire dataset until the test phase. Models with a large number of measures compared with events (responder or non-responder) risk overfitting and demonstrate poor generalisability to novel datasets. Our dataset contained 35 potential features and therefore was at risk of overfitting. To address this issue, we reduced the number of data-dimensions via feature selection, employed cross-validation and used an automated tuning of the regularisation parameter ‘C’. However, while these techniques may ameliorate some of the risk of overfitting, a future study with larger sample size, or independently collected datasets, would take the next steps to externally validate the brain-behaviour model and allow assessment of generalisability.

A key feature of support vector machines is that they fit high-dimensional discriminatory planes between multiple measures to predict an outcome. This multivariate approach affords greater sensitivity in distinguishing between non-separable distributions along a single dimension. The additional use of a non-linear kernel also enables us to capture relationships between highly disparate biological features which often demonstrate non-linear profiles and as a result predict changes in breathlessness. Although this technique leads to less intuitive interpretations of feature weightings, the methods used are the first to successfully demonstrate a relationship between breathlessness expectation related brain activity and changes to reported breathlessness over pulmonary rehabilitation. To reconcile these challenges and move towards eventual clinical application, we suggest that this model form the basis for further studies scrutiny via first an external validation dataset and then further interventional studies.

While larger sample sizes are now required to translate these mechanistic models into clinical relevance, the data provides evidence that breathlessness expectation related brain activity at baseline strongly influences how patients respond to treatment in a predictable manner.

## Conclusions

This study offers the first steps towards brain-based predictive biomarkers for pulmonary rehabilitation outcome. We have shown that models including objective brain markers of breathlessness-expectation are able to predict, for the first time, which patients will have clinically important improvements in breathlessness over pulmonary rehabilitation. Such models could provide new insights into the mechanisms by which breathlessness may be targeted, paving the way for targeted behavioural and pharmacological interventions.

## Data Availability

Data are available on reasonable request.
